# Both psychological factors and physical performance are associated with fall-related concerns

**DOI:** 10.1007/s40520-017-0882-9

**Published:** 2017-12-20

**Authors:** Mascha Pauelsen, Lars Nyberg, Ulrik Röijezon, Irene Vikman

**Affiliations:** Division of Health and Rehabilitation, Department of Health Sciences, Luleå University of Technology, Luleå, Sweden

**Keywords:** Aged, Fall-related concerns, Morale, Ambulation, Self-efficacy

## Abstract

**Background:**

Fall-related concern strongly correlates to activity avoidance in older people. In this complex phenomenon, different terminology and instruments are often used interchangeably. Three main concepts make up fall-related concerns: fear of falling, consequence concern, and falls self-efficacy. It is suggested that fall-related concerns are mediated by psychological and physical factors.

**Aims:**

Our aims were to describe the prevalence of fall-related concerns and find explanatory factors for its most studied concept—falls self-efficacy—in an older population.

**Methods:**

We executed a cross-sectional study on a random sample of 153 community-dwelling older people (70 years or older). We used validated and reliable instruments as well as structured interviews to gather data on the three concepts of fall-related concerns and possible mediating factors. We then calculated descriptive statistics on prevalence and regression models for the total group, and men and women, separately.

**Results:**

70% of the total sample (80% of women and 53% of men) reported at least one of the three concepts of fall-related concern. For the total sample, fear of falling, morale, and physical performance were associated factors with falls self-efficacy. For women, the number of prescription medications was added. For men, physical performance and concerns for injury were associated.

**Conclusion:**

Fall-related concern is prevalent in large proportions with higher prevalence for women than for men. Important factors are fear of falling, morale, and physical performance. Gender differences in the emergence and variance of fall-related concern and the relation between physical performance and fall-related concern should be targeted in future research endeavors.

## Introduction

Ageing is associated with a decline in sensory-motor systems [1–4]. While this happens, reaction times increase and the risks of falls and developing fall-related concerns (FrC) rise [5–12]. With a clear correlation between FrC and activity avoidance, FrC poses a threat to the general well-being of older adults [13].

The area has been studied since the 1980s [7, 14] and has evolved to become an important research area in relation to active ageing. FrC have been described under various names: fear of falling (FoF), falls self-efficacy (FsE) or lack of balance confidence, and consequence concerns (CC) [15]. Researchers in the field have shown difficulty in separating these different concepts [16]. Concept descriptions range from being afraid that one will fall, to having concerns about a range of factors and consequences related to a fall. Often, older adults express concerns about not being able to get up or needing assistance in activities of daily living after the fall, in addition to fear of the fall itself [17]. The term *fall-related concerns* includes all previously named concepts: FoF, FsE, and CC [18].

Up to 55% of the older adults living in the community are reported to suffer from FrC [19]. The prevalence increases with age and is higher for women than for men [8]. Possible underlying mechanisms for gender differences have been discussed [20], but not much is known. FrC relates strongly to activity avoidance and thus to a higher risk of falls [21–26]. These prevalence numbers show a large variability between studies.

As mentioned, however, the understanding of the field is hampered by differences in conceptualization and terminology [27]. This is likely to be one major explanation behind the large variation in reported prevalence rates, associated risk indicators, and the rather weak prevention evidence. In many studies, FsE is measured as a representation of FrC while naming it FoF. As mentioned above, both FsE and FoF are concepts within fall-related concern. The instrument used to measure FsE (Falls Efficacy Scale-International), is a widely recognized, reliable, and validated questionnaire, which is easy to administer and has good psychometric properties [27].

Traditionally, FrC are seen as a result of a fall or near-fall experience, leading to low balance confidence, leading to activity avoidance, leading to functional decline, and leading to a greater risk of falls. However, FrC are prevalent among older adults without fall experiences, as well [26, 28, 29]. An independent association, controlled for the effect of postural instability, between FrC and fall risk remains to be demonstrated. A more multifactorial concept has been suggested in which the relation between falls and FrC would be mediated by postural stability, and that postural stability and fear influence one another, as suggested via the appraisal of one’s own ability [15]. Even though there are some studies describing altered gait patterns in elderly and reduced balance in people with FrC [28, 29], studies on how physical performance is associated to FrC are scarce. Differences in postural control strategies when standing at various heights have been demonstrated [28], and decreased medio-lateral stability during transition from sit to walk in people having FrC, as compared to those who had not, has been shown [30]. Another aspect of this conceptualization is the mediating effect of psychological factors on FrC. Psychological variables, such as depressive symptoms and outlook on life, have shown to contribute to variance in FrC [31]. Outlook on life as one ages is often described and measured as morale [32].

### Aims

We set out to take the understanding of the concept of FrC among older adults a few steps further. We will do this by describing the prevalence of FrC and its concepts among older adults. We will also attempt to describe how the degree of one of its concepts, namely FsE, can be related to possible mediating factors, such as mobility-related physical performance, previous fall experiences, morale, and other factors known to be related. We will also present descriptions and models for men and women separately.

## Methods

We executed a cross-sectional study, based on data from a structured interview and functional tests in a random community-dwelling sample of people aged 70 or more, collected during home visits.

### Population and sampling

We randomly selected 300 + 200 persons in two batches (minus 9 doubles) from the Swedish Population Register (Statens Personadressregister, SPAR). Inclusion criteria were living in the community of a specific Northern Swedish municipality and 70 years of age or older. The one exclusion criaterion was: not having the cognitive ability to make and keep our visit appointment themselves. Of the selected 491 persons, 22 lived in sheltered accommodations and were excluded. We were unsuccessful in finding telephone numbers for 44 persons, leaving us with 424 included prospective participants who received an invitation letter describing the study. We then proceeded to call them by phone to invite them to participate in the study. Between selection and approach, 1 person had deceased and we could not reach 62 persons after four tries. Of the remaining 362, 153 (42%) participated. We present their characteristics in Table 1.

**Table 1:** Sample characteristics

Characteristics	Total	Women	Men
Participants, *n* (%)	153	96 (63)	57 (37)
Age, mean ± SD	78.0 ± 6.2	78.6 ± 6.3	77.5 ± 6.1
Living alone, *n* (%)	64 (42)	47 (49)	17 (30)^§^
Has home help services, *n* (%)	13 (9)	9 (9)	4 (7)
Life space, mean ± SD	68.6 ± 22.9	63.2 ± 22.8	77.7 ± 20.1*
Number of prescription medications, mean ± SD	3.4 ± 2.7	3.6 ± 2.8	3.2 ± 2.6
≥ 1 falls past 6 months, *n* (%)	45 (29)	23 (24)	22 (39)
≥ 1 falls past month, *n* (%)	24 (16)	8 (8)	3 (5)
Mini-mental test (0–30), mean ± SD	28 ± 2	28 ± 2	28 ± 2
Short physical performance battery (0–12), mean ± SD	9.4 ± 2,8	9.1 ± 2.9	9.8 ± 2.6
Balance, median (Q1, Q3)	4 (3, 4)	4 (2, 4)	4 (3, 4)
Walking, median (Q1, Q3)	4 (3, 4)	4 (3, 4)	4 (3, 4)
Chair stands, median (Q1, Q3)	3 (2, 4)	3 (2, 4)	3 (2, 4)
Philadelphia geriatric scale of morale (0–17), mean ± SD	13.0 ± 2.9	12.6 ± 3.1	13.7 ± 2.6*

*Significant difference between women and men to the 0.05 level, using the independent samples *t* test

^§^Significant difference between women and men to the 0.05 level, using the *χ* ^2^ test

### Instruments and procedures

Four licensed physiotherapists who had experience of each of the instruments collected the data during the home visits. Before the visits, we strengthened inter-rater reliability through pilot testing and discussions about testing procedures and results from the pilots.

Besides collecting data on demographics, co-habiting situation, mobility aids, and home help, we also asked the participants about their use of prescription medication. The latter, by together with the participant, going through their medication list, which they acquired from their apothecary. Fall history during the last 6 months was asked for, for which the definition of a fall was: “an unexpected event in which the participant comes to rest on the ground, floor, or lower level” [33].

FsE was measured with the Falls Efficacy Scale-International, Swedish version (FES-I), which is a questionnaire instrument with 16 items [34]. Participants are asked to assess how concerned they are about falling while doing certain activities at home, outdoors, and socially. Items are answered on a Likert scale of 1 (not at all concerned) to 4 (very concerned). Scores range from 16 to 64. FoF was assessed by a single-item question “Are you afraid of falling?” using a Likert scale of 1 (no, not afraid) to 4 (yes, very much) [35]. To assess CC, we used the question “If you were to fall, would you be concerned about….?”, which was answered on a Likert scale of 1 (no) to 4 (Yes, very concerned) [26]. The Mini-Mental State Examination (MMSE) provides a brief screening test of cognitive impairment [36]. Scores range from 0 to 30 (no cognitive impairment). The Swedish version of Life Space Assessment [37] includes six levels of life space, ranging from the person’s bedroom to places beyond the person’s home town. The total score can range from 0 (totally confined to bed) to 120 (independent, with daily out-of-town mobility). The Short Physical Performance Battery (SPPB) consists of functional tests concerning standing balance, walking, and chair stands [38]. Scores range from 0 to 12. The Philadelphia Geriatric Center Morale Scale (PGCM) consists of 22 yes or no questions about interpersonal and intrapersonal aspects of ageing, surrounding three main factors: agitation, attitude toward own ageing, and lonely dissatisfaction [39]. Scores range from 0 to 22. For all instruments mentioned, a higher score means a larger amount or presence of the measured attribute.

Since FoF, FsE, and CC all seem to be aspects of FrC, for descriptive purposes, we have assumed that a person reporting any of the three aspects (so either heightened FoF or CC, or declined FsE) is, in fact, reporting that they experience fall-related concerns.

### Analyses

Descriptive statistics were calculated, and, as indicated in text and tables, independent samples *t* test, *χ* ^2^ test, risk ratio, as well as 95% confidence intervals were used to do this. We then applied a principle component analysis (PCA) with varimax rotation to reduce dimensions. Variables that entered the PCA were: age, sex, living alone, having home help, self-rated health, number of medicines, having musculoskeletal pain, falls during the past months, falls during the past 6 months, FoF, CC-I, CC-L, CC-H, CC-B, MMSE, SPPB, and PGCM. We then proceeded with multiple regression analyses to model associations to prevalence and degree of fall-related concern. We used SPSS (IBM SPSS statistics 23) for all statistical calculations. We checked the data and models for parametric properties to ensure statistical validity.

### Ethical considerations

Informed consent was obtained from all individual participants included in the study. This study design was approved by the Regional Ethical Review Board in Umeå, Sweden (ref no. 2015-182-31) and with the 1964 Helsinki declaration and its later amendments or comparable ethical standards.

## Results

### Participant characteristics and FrC prevalence

The characteristics of our participants, who all lived in the community, are shown in Table 1.

The prevalence for each of the concepts is significantly different between men and women for all concepts apart from CC-H (needing more help after a potential fall), as shown in Table 2.

**Table 2:** Descriptive statistics for FES-I, FoF, and CC

	Total *n* = 153	Women *n* = 96	Men *n* = 57	*p* value
	Mean ± SD	Mean ± SD	Mean ± SD	
FES-I	22 ± 5.8	23 ± 5.6	20 ± 5.7	0.007

	% (95% CI)	% (95% CI)	% (95% CI)	Risk ratio (95% CI)
High FES-I (≥ 23)	39 (31–47)	48 (38–58)	23 (12–34)	2.1 (1.25–3.54)*
Fear of falling (FoF)^a^	38 (30–46)	50 (40–60)	18 (8–28)	2.9 (1.57–5.18)**
Concerns about injury from a fall (CC-I)^a^	51 (43–59)	60 (50–70)	35 (23–47)	1.7 (1.17–2.54)*
Concerns about not getting up after a fall (CC-L)^a^	31 (24–38)	49 (39–59)	12 (4–20)	3.4 (1.63–7.06)**
Concerns about needing s(more) help after a fall (CC-H)^a^	25 (18–32)	30 (21–39)	16 (6–26)	1.9 (0.98–3.75)
Concerns about being a burden after a fall (CC-B)^a^	26 (19–33)	33 (24–42)	14 (5–23)	2.4 (1.18–4.79)*
Reporting some form of FrC^b^	70 (63–77)	80 (72–88)	53 (40–66)	1.5 (1.17–1.99)**

*Significant to the 0.05 level

**Significant to the 0.001 level

^a^Questions were answered on a 1–4 scale where 1 = no and 4 = very much. Proportions shown are those who answered > 1. The difference between men and women is reported with its risk ratio

^b^Proportions are those who reported an FES-I score of ≥ 23 [40] and/or answered > 1 on one or more of the FoF and CC questions. The difference between men and women is reported with its risk ratio

Looking at the associations between the three FrC concepts, we see the following: each concept is low-to-moderately associated with each of the other two concepts. The highest association exists between CC and FoF (*φ* = 0.51) followed by the association between FoF and FsE (*φ* = 0.46) and the lowest exists between FsE and CC (*φ* = 0.30).

### Models

The PCA showed us that our data consisted of four components. The first one consisting of: FoF and the CCs; the second one consisting of: age, SPPB, living alone and MMT; the third consisting of: self-rated health, number of medicines, musculoskeletal pain and PGCM; and the fourth consisting of: falls (1 and 6 months). Based on the component division, the scores within them, the existing literature, and our aims, we decided to eliminate only a few of the variables. The variables used in the regression modelling are presented in Table 3. The stepwise entry for the model then showed which of the remaining variables were of importance for the explanation of FES-I as the dependent variable.

**Table 3:** Forward stepwise multiple regression models of variables associated with FES-I, for total sample, women, and men

Independent variables	Coefficient (95% CI)	Standardized coefficient	*p* value	Variance inflation factor
For total sample (*n* = 153)*				
Constant	32.837 (29.081–36.594)			
Fear of falling	2.792 (2.006–3.578)	0.434	< 0.001	1.170
SPPB	− 0.605 (− 0.843 to − 0.367)	− 0.303	< 0.001	1.120
PGCM	− 0.531 (− 0.764 to − 0.764)	− 0.272	< 0.001	1.126
For women (*n* = 96)**				
Constant	29.021 (24.315–33.727)			
Fear of falling	2.558 (1.689–3.428)	0.437	< 0.001	1.140
PGCM	− 0.498 (− 0.775 to − 0.221)	− 0.278	0.001	1.138
Number of medications	0.375 (0.101–0.649)	0.203	0.008	1.049
SPPB	− 0.360 (− 0.636 to − 0.085)	− 0.196	0.011	1.065
For men (*n* = 57)***				
Constant	34.684 (30.183–39.186)			
SPPB	− 1.320 (− 1.710 to − 0.929)	− 0.607	< 0.001	1.026
CC-injury	3.338 (1.764–4.912)	0.381	< 0.001	1.026

Dependent variable: FES-I

Variables included in the modelling: fear of falling, SPPB, PGCM, number of medications, age, living alone, self-rated health, previous falls, MMSE, CC-I, CC-L, CC-H, and CC-B. As well as sex for the total sample

*Model adjusted *R* ^2^: 0.54, *p* < 0.001, Durbin–Watson statistic: 1.903

**Model adjusted *R* ^2^: 0.55, *p* < 0.001, Durbin–Watson statistic: 2.103

***Model adjusted *R* ^2^: 0.57, *p* < 0.001, Durbin–Watson statistic: 1.887

The reported sex differences in the prevalence of reduced falls self-efficacy called for a multiple regression model for both sexes separately, as well as for the whole sample. We present the models in Table 3 with their respective diagnostics. All models include physical performance (SPPB).

## Discussion

Our study describes a high prevalence of older adults suffering from any form of FrC as well as each of its three concepts, with significant gender differences. We then went on to show that the three FrC concepts describe related phenomena, but are not the same thing. Finally, we created regression models, which show that morale, physical performance, and FoF are important factors to explain FsE to a high degree for the entire group, but that there are important differences in these models when separating men and women.

The prevalence we found for FsE was similar to that found in the previous studies [41–43]. However, when taking into account that FrC consists of three different concepts, the numbers change dramatically. The proportion of participants who reported having any one of the aspects was much higher than the previous literature, 70% for the entire sample with women having 1.5 times as much risk of developing FrC than men. The possible effects of strong gender roles when it comes to FrC were discussed [20], but, to our knowledge, never investigated. Our results show a strong need for research on gender differences in the emergence of FrC.

Our material showed moderate correlations between the three FrC concepts, indicating that the concepts are relating but different phenomena. These associations and the differing prevalences we found for each concept, in combination with the different ‘definitions’ and names of FrC used in the field [27], indicates, once again, the importance of reaching a consensus about how to define and model FrC. This separation of concepts is of clinical importance, as well. A person might experience a lowered FsE while not experiencing FoF, or they might experience a heightened CC and FoF but no change in FsE. Especially older adults who experience the latter would not be seen if only FES-I was used as a measurement of FrC.

Physical performance is an important factor in each of the models and two of our models include both physical performance and psychological factors as important associating factors for FsE, as was to be expected based on the background conceptualization of FrC. This is in line with an earlier published conceptualization, presenting that the belief that one would probably fall during a specific activity is mediated by fear of falling and actual balance performance, and vice versa [15]. This implies that in many cases, FrC is partially based on actual balance ability, which should not be overlooked in a clinical setting. Balance is, however, a complex activity and SPPB is an instrument consisting of functional tests. This makes it difficult to observe which specific aspects of balance performance are influencing the SPPB score the most and thus correlate to FrC the strongest. In the comprehensive design of the current study, there was no room to test and evaluate postural control in a more detailed way. Kinematic and kinetic testing targeting specifically older adults’s postural control in relation to FrC’s three concepts is recommended to further unravel the mediating role of physical performance on FrC.

Morale, which is measured with the PGCM, can be defined as “…a multidimensional concept, often defined as a future-oriented optimism or pessimism regarding the problems and opportunities associated with ageing” [32]. It is associated with variables such as family support, physical health, depression, and ADL independence while it is not associated with variables such as age, sex, cognitive impairment, or marital status [44]. This, in combination with its good reliability and validity, leads us to accept the PGCM instrument as a very appropriate one to measure psychological factors in relation to FrC. We recommend that this instrument is included in future research in the field. Our findings suggest that, at least for the group as a whole and for women, a generally more pessimistic outlook on life while ageing is associated with higher FrC scores.

Our regression model for FsE in men did not include FoF or morale, but added a concern for injury to physical performance. It is unclear if this concern is a physical or social concern. By that, we mean whether the concern is for the immediate discomfort that comes with an injury or if the concern is for the consequences of the injury, i.e., becoming less independent, forced to stop doing chores around the home or garden, etc. Nevertheless, we see a clear gender-based difference between the models, which needs to be investigated further.

It is important to note that none of our final regression models incorporated previous falls, while it was an included variable during the modelling process. This could mean that the association between FrC and previous falls found in earlier studies [45–48] might be better explained through another mediating variable—likely physical performance.

### Strengths and limitations

We chose to focus on community-dwelling older adults and created a population sample for that purpose. The group of older adults living in sheltered housing tends to be quite different from community-dwelling older adults when it comes to possible risk factors for falls and FrC. We, therefore, deem it important that this group is investigated as separately.

When comparing our sample to the population, we had a similar age distribution (mean 78.0 years in the sample versus 78.1 in the population), but a tolerable overrepresentation of women (63 versus 55%). In comparison with two relevant population studies, we found that our age difference adjusted sample mean life space was included in the 95% confidence interval of a recent Swedish study [49]. However, the SPPB mean (likewise adjusted) turned out to be just above that of a Spanish study [50], indicating a slightly more fit sample.

For all aspects of this study, we have to keep in mind that it had a cross-sectional design and, thus, cannot report anything about actual cause and effect. However, when we consider these associations in relation to the literature, models of possible mediation do seem to appear. These should be investigated more with the help of a longitudinal design.

## Conclusion

Our study reveals that fall-related concern is prevalent in large proportions of the older population with a higher prevalence for women than for men. The major factors associating with falls self-efficacy in women are fear of falling, morale, number of medications, and physical performance, while the model for men contained physical performance and injury concerns only. Gender differences in the emergence and variance of fall-related concerns and the relation between physical performance and fall-related concerns should, therefore, be targeted in future research endeavors.
